# P-762. Comparative Analysis Of Inpatient mortality, 30-Day And 60-Day Re-admissions in Diabetic Ketoacidosis (DKA) Patients with Urinary Tract Infections And Pneumonia

**DOI:** 10.1093/ofid/ofaf695.973

**Published:** 2026-01-11

**Authors:** Chinar Singh, Hamza Khan, Danish Saeed, Magyury Gomez, Amogh Killedar, Martin Andrew

**Affiliations:** HCA Healthcare Westside/Northwest, Plantation, Florida; HCA Healthcare Westside/Northwest, Plantation, Florida; HCA Healthcare Westside/Northwest, Plantation, Florida; HCA Healthcare Westside/Northwest, Plantation, Florida; HCA Healthcare Westside/Northwest, Plantation, Florida; HCA Healthcare Northwest, Plantation, Florida

## Abstract

**Background:**

Diabetic ketoacidosis (DKA), a life-threatening diabetes complication, is often triggered by infections— which cause nearly half of cases —often pneumonia and UTIs. Infections worsen DKA severity, prolong hospital stays, and increase mortality. Despite this, few studies compare DKA outcomes by infection type. This study analyzes infection-related DKA, focusing on UTIs and pneumonias, and evaluates their impact on in-hospital mortality and 30-/60-day readmission rates.

**Results:**

**Figure:**
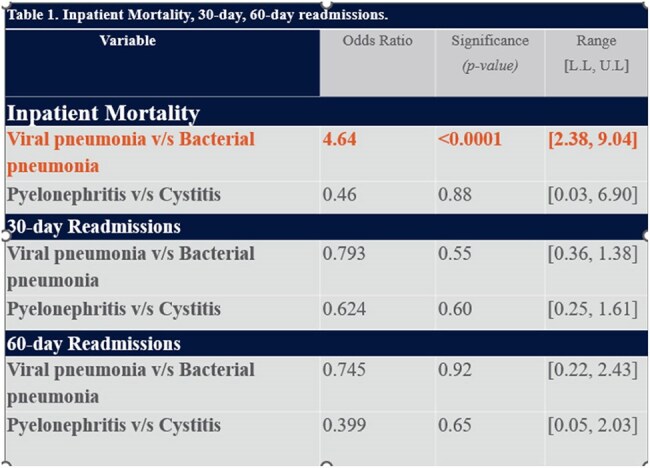

Results for mortality , 30-/60-day readmission rates

**Figure:**
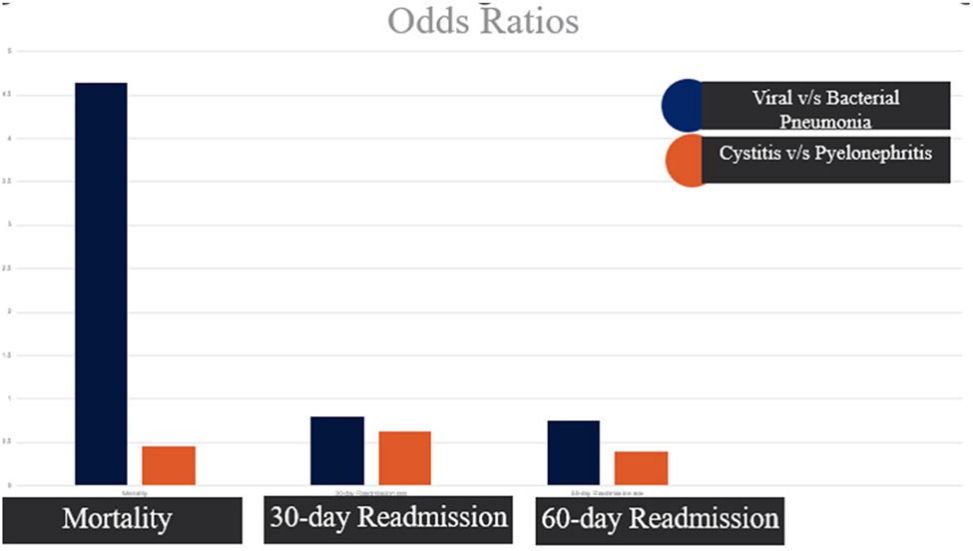
Graph comparing ODDS ratio among infections for mortality 30-/60-day readmission rates

**Methods:**

This retrospective cohort study included adults (≥18 years) admitted with DKA and a concurrent infection—pyelonephritis, cystitis, bacterial viral pneumonia at HCA East Florida facilities from January 2020 to December 2023. Patients were excluded if they were immunocompromised, pregnant, had active cancer, or multiple infections. Patients who died during index hospitalization were excluded from readmission analyses. Logistic regression assessed outcomes, adjusting for age, sex, race and infection type.

**Results:**

Among 3,276 patients, 474 (14.47%) died or were discharged to hospice. During index hospitalization, Viral pneumonia was associated with significantly higher mortality compared to bacterial pneumonia (OR 4.64; P < .0001) whereas no mortality difference was noted between pyelonephritis and cystitis (P = .8809). Of survivors, 583 (21.1%) and 206 (27.0%) were readmitted within 30 and 60 days respectively. No significant differences in 30 or 60-day re-admissions were found between infection types.

**Conclusion:**

Viral pneumonia, likely driven by COVID-19, was linked to significantly higher DKA-related mortality, unlike differences seen in UTI types. While infection type didn’t influence readmission risk, it clearly shaped in-hospital outcomes—underscoring the need for infection-specific strategies in managing DKA, especially in the post-COVID era. These findings call for continued research into modifiable risk factors and demand heightened clinical vigilance during initial hospitalization, particularly when viral pneumonia is involved.

**Disclosures:**

All Authors: No reported disclosures

